# Nutrient generation and retrieval from the host cell cytosol by intra-vacuolar *Legionella pneumophila*

**DOI:** 10.3389/fcimb.2014.00111

**Published:** 2014-08-26

**Authors:** Christopher T. D. Price, Ashley M. Richards, Yousef Abu Kwaik

**Affiliations:** Department of Microbiology and Immunology and Center for Predictive Medicine, College of Medicine, University of LouisvilleLouisville, KY, USA

**Keywords:** Legionnaires' disease, proteasomes, ubiquitin, slc transporter, AnkB, F-box, farnesylation

Microbial acquisition of nutrients *in vivo* is a fundamental aspect of infectious diseases, and is a potential target for anti-microbial therapy. Part of the innate host defense against microbial infection is nutritional restriction of access to sources of host nutrients (Abu Kwaik and Bumann, 2013; Eisenreich et al., 2013). Despite this host nutritional restriction, there has been a long held presumption that the host cell cytosol has sufficient nutrients for any intracellular pathogen, although many bacteria fail to grow in the host cytosol if they are microinjected (Goetz et al., 2001). However, recent studies on the two intra-vacuolar pathogens *Anaplasma phagocytophilum* (Niu et al., 2012) and *Legionella pneumophila* (Price et al., 2011) and the cytosolic pathogen *Francisella tularensis* (Steele et al., 2013) have clearly shown that the levels of amino acids in the host cell cytosol are below the threshold sufficient to meet the tremendous demands for carbon, nitrogen and energy to power the robust intracellular proliferation of these pathogens (Abu Kwaik and Bumann, 2013). Therefore, these intracellular pathogens have evolved with efficient strategies to boost the levels of host amino acids to meet their demands for higher levels of carbon, nitrogen and energy sources (Abu Kwaik and Bumann, 2013; Fonseca and Swanson, 2014). There is an emerging paradigm of specific microbial strategies that directly trigger the host cell to boost the cellular levels of essential microbial nutrients, and this paradigm has been designated as “nutritional virulence” (Abu Kwaik and Bumann, 2013). This opinion article is focused on nutritional virulence of *L. pneumophila*.

In the aquatic environment, *L. pneumophila* proliferates within protozoa, which impact bacterial ecology and pathogenicity (Al-Quadan et al., 2012). Upon transmission to humans, *L. pneumophila* proliferates in alveolar macrophages within the *Legionella*-containing vacuole (LCV) that is ER-derived and evades lysosomal fusion (Figure 1). Within both evolutionarily distant host cells, the Dot/Icm type IV secretion system of *L. pneumophila* injects ~300 protein effectors (Zhu et al., 2011; Luo, 2011a) that govern biogenesis of the LCV and modulate a myriad of cellular processes to enable intra-vacuolar proliferation (Figure 1) (Luo, 2011b; Richards et al., 2013).

**Figure 1:**
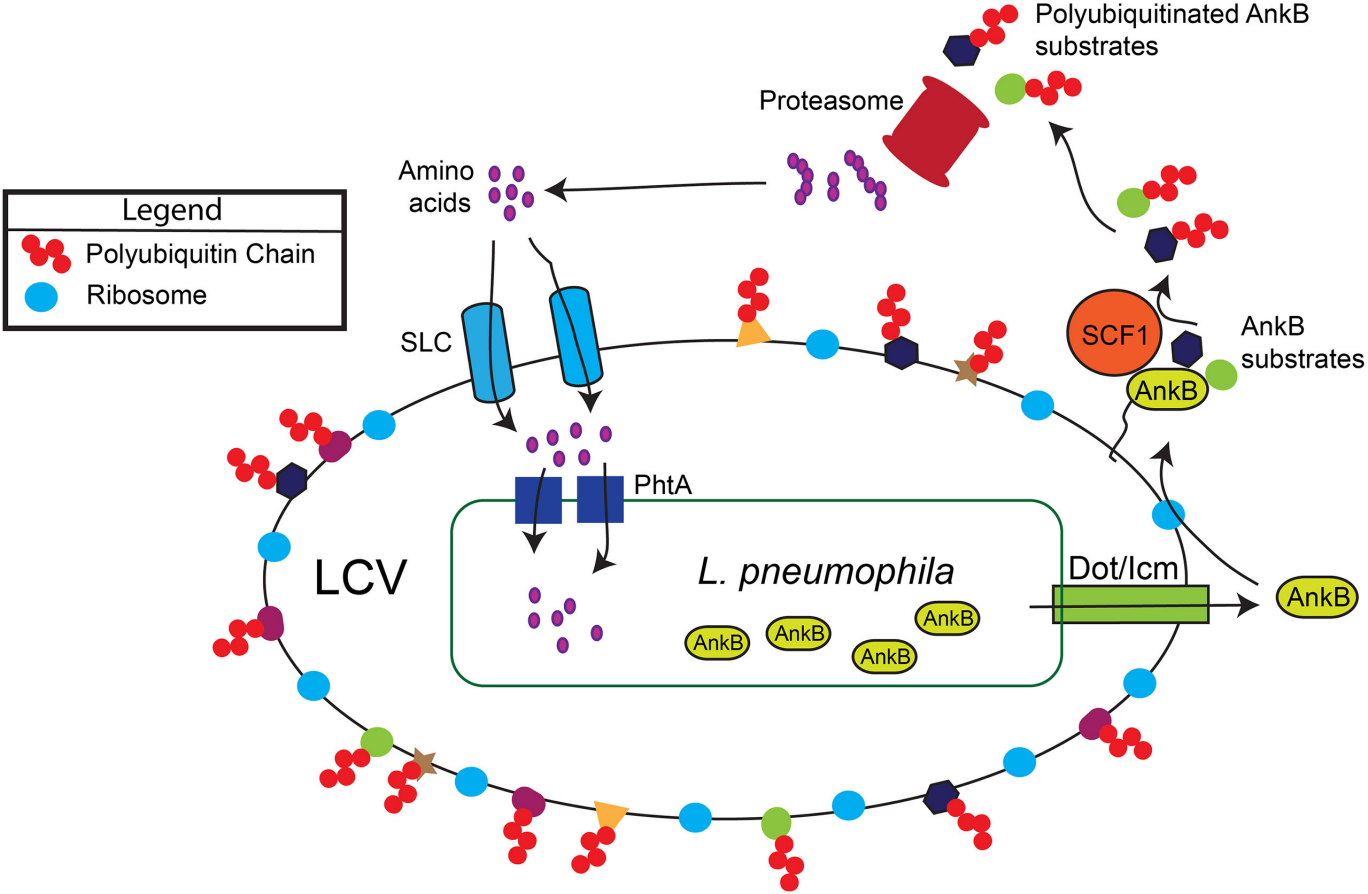
**Generation of a surplus of host amino acids by *L. pneumophila* and their import into the LCV**. The AnkB effector is translocated into macrophages and amoebae by the Dot/Icm type IV secretion system of *L. pneumophila*. AnkB is immediately farnesylated and anchored into the cytosolic face of the LCV membrane where it interacts with the eukaryotic SCF1 ubiquitin ligase complex. The AnkB effector functions as a platform for the docking of Lys ^48^-linked polyubiquitinated proteins to the LCV that are subsequently degraded by the proteasome, which generates a surplus of cellular amino acids above the threshold necessary for intra-vacuolar proliferation. The amino acids are likely imported into the LCV through various host SLC amino acid transporters in the LCV membrane, but the identity of the transporters is still to be determined. The amino acids are acquired by *L. pneumophila* through numerous ABC transporters and amino acid permeases such as the threonine transporter PhtA.

Amino acids are the main sources of carbon, nitrogen and energy for *L. pneumophila*, which metabolizes them through the TCA cycle (Pine et al., 1979), but also metabolizes minor amounts of glucose *in vitro* using the Entner-Doudoroff pathway (Eylert et al., 2010; Price et al., 2011). Although *L. pneumophila* utilizes amino acids as the main sources of carbon and energy, the pathogen is auxotrophic for seven amino acids (Cys, Met, Arg, Thr, Val, Ile, and Leu) (Eylert et al., 2010; Price et al., 2014). Remarkably, there is a high level of synchronization in amino acids auxotrophy between *L. pneumophila* and its host cells, which has likely played a factor in nutritional evolution of *L. pneumophila* as an intra-vacuolar pathogen (Price et al., 2014).

Interestingly, intra-vacuolar *L. pneumophila* up-regulates its own amino acids transporters, indicating increased demands for amino acids in the intra-vacuolar environment (Bruggemann et al., 2006; Faucher et al., 2011; Eisenreich et al., 2013). Since the generation time of intra-vacuolar *L. pneumophila* is ~40 min, this organism requires high levels of amino acids to be imported from the host cytosol into the LCV lumen (Schunder et al., 2014). A long-held presumption has been that the host cell cytosol is rich in nutrients for invading pathogens.

However, recent studies clearly indicate that the basal levels of host cellular amino acids are below the threshold sufficient for the robust intra-vacuolar proliferation of *L. pneumophila* (Sauer et al., 2005; Wieland et al., 2005) To achieve that needed threshold, *L. pneumophila* promotes host proteasomal degradation (Price et al., 2011) of LCV-decorated polyubiquitinated proteins (Dorer et al., 2006; Price et al., 2009, 2011; Lomma et al., 2010) mediated by the AnkB effector.

Within human macrophages and amoeba, the AnkB translocated effector of *L. pneumophila* strain AA100/130b is localized to the cytosolic face of the LCV membrane through host-mediated farnesylation of its C-terminal CaaX motif (Figure 1) (Price et al., 2010; Al-Quadan et al., 2011; Al-Quadan and Kwaik, 2011). On the LCV membrane, AnkB interacts with the host SCF1 ubiquitin ligase (Figure 1) (Bruckert et al., 2014). As a *bona fide* F-box effector (Ensminger and Isberg, 2010; Lomma et al., 2010; Price and Abu Kwaik, 2010), AnkB triggers decoration of the LCV with Lys ^48^-linked polyubiquitinated proteins that are targeted for proteasomal degradation (Figure 1) (Price et al., 2011). The metabolomic profile of *L. pneumophila-infected* amoeba and human cells have shown an AnkB-dependent dramatic rise in the levels of all cellular amino acids (Price et al., 2011), and this is initiated rapidly upon bacterial attachment to the macrophage plasma membrane (Bruckert et al., 2014). Importantly, inhibition of host proteasomal degradation abolishes intracellular proliferation of *L. pneumophila* strains AA100/130b and Philadelphia (Dorer et al., 2006; Price et al., 2011). The *L. pneumophila*-generated surplus of host cell amino acids may explain the lack of an intracellular defect for the lysine and tryptophan auxotrophic mutants of *L. pneumophila* (Mintz et al., 1988; Ensminger et al., 2012).

Loss of AnkB in two independent isolates (AA100 and Paris) results in varying degrees of failure in intra-vacuolar proliferation and attenuation in the mouse model of Legionnaires' disease (Al-Khodor et al., 2008; Lomma et al., 2010). These defects for the AA100 strain are totally overcome upon supplementation of a mixture of amino acids in macrophages, amoeba and in the mouse model, similar to genetic complementation (Price et al., 2011). Importantly, silencing of the host SCF1 ubiquitin ligase, interference with Lys^48^-linked polyubiquitination, or inhibition of the host proteasomes block intra-vacuolar proliferation of *L. pneumophila*, but the block is relieved upon supplementation of an excess mixture of amino acids (Price et al., 2009, 2011). Surprisingly, the intra-vacuolar proliferation defect of the *ankB* mutant is rescued by many individual amino acids, such as Cys, Ala or Ser, which are essential or metabolically favorable for *L. pneumophila* (Pine et al., 1979). Interestingly, although Gln is the most abundant amino acid in human cells, supplementation of infected hMDMs with excess Gln alone efficiently rescues the *ankB* mutant (Price et al., 2011), while Glu is a major source of carbon and energy *in vitro* (Pine et al., 1979). These findings indicate that the basal levels of cellular amino acids are below the threshold sufficient for intra-vacuolar proliferation of *L. pneumophila*.

Remarkably, pyruvate or citrate supplementation is as effective as amino acids in rescuing the intra-vacuolar growth defect of the *ankB* mutant, which indicates that the LCV is capable of importing these two substrates that can feed the TCA cycle, in addition to the documented reliance of intra-vacuolar *L. pneumophila* on amino acids (Schunder et al., 2014). In addition, *L. pneumophila* utilizes glucose through the Entner-Doudoroff pathway, which is required for proliferation within the amoeba host (Eylert et al., 2010). During inflammation, macrophages undergo up-regulation of glucose uptake and anaerobic glycolysis (Warburg-effect), which generates additional pyruvate (Eisenreich et al., 2013), and it is likely that both glucose and pyruvate are imported by the LCV. In addition, *L. pneumophila*-infected macrophages exhibit a pro-inflammatory phenotype. Taken together, it is likely that a multi-prong nutritional virulence strategy is utilized by *L. pneumophila* to generate and retrieve a diversified portfolio of sources of carbon and energy from the host cell. The host solute carrier (SLC) family of membrane proteins (Cedernaes et al., 2011; Schioth et al., 2013) that transport various compounds, including amino acids, TCA intermediates, glucose, lipids, and drugs are likely to be involved in import of various compounds by the LCV membrane (Figure 1) (Wieland et al., 2005). Future studies should unravel the host metabolites and the mechanism of their import into the LCV lumen, and subsequently by the bacterial membrane. Deciphering microbial nutrition and metabolism *in vivo* is essential for our understanding of host-microbe interaction, and nutrient retrieval strategies by intracellular pathogens are potential targets for therapy.

## Conflict of interest statement

The authors declare that the research was conducted in the absence of any commercial or financial relationships that could be construed as a potential conflict of interest.
